# Novel Use of the Nintendo Wii Board for Measuring Isometric Lower Limb Strength: A Reproducible and Valid Method in Older Adults

**DOI:** 10.1371/journal.pone.0138660

**Published:** 2015-10-07

**Authors:** Martin Gronbech Jorgensen, Stig Andersen, Jesper Ryg, Tahir Masud

**Affiliations:** 1 Department of Geriatrics, Aalborg University Hospital, Aalborg, Denmark; 2 Department of Geriatrics, Odense University Hospital, Odense, Denmark; 3 Institute of Clinical Research, University of Southern Denmark, Odense, Denmark; 4 Health care for Older People, Nottingham University Hospital NHS Trust, Nottingham, Notts, United Kingdom; 5 Department of Clinical Medicine, Aalborg University, Aalborg, Denmark; Universidad Europea de Madrid, SPAIN

## Abstract

**Background:**

Portable, low-cost, objective and reproducible assessment of muscle strength in the lower limbs is important as it allows clinicians to precisly track progression of patients undergoing rehabilitation. The Nintendo Wii Balance Board (WBB) is portable, inexpensive, durable, available worldwide, and may serve the above function.

**Objective:**

The purpose of the study was to evaluate (1) reproducibility and (2) concurrent validity of the WBB for measuring isometric muscle strength in the lower limb.

**Methods:**

A custom hardware and software was developed to utilize the WBB for assessment of isometric muscle strength. Thirty older adults (69.0±4.2 years of age) were studied on two separate occasions on both the WBB and a stationary isometric dynamometer (SID). On each occasion, three recordings were obtained from each device. For the first recording, means and maximum values were used for further analysis. The test-retest reproducibility was examined using intraclass correlation coefficients (ICC), Standard Error of Measurement (SEM), and limits of agreement (LOA). Bland-Altman plots (BAP) and ICC’s were used to explore concurrent validity.

**Results:**

No systematic difference between test-retest was detected for the WBB. ICC within-device were between 0.90 and 0.96 and between-devices were from 0.80 to 0.84. SEM ranged for the WBB from 9.7 to 13.9%, and for the SID from 11.9 to 13.1%. LOA ranged for the WBB from 20.3 to 28.7% and for the SID from 24.2 to 26.6%. The BAP showed no relationship between the difference and the mean.

**Conclusions:**

A high relative and an acceptable absolute reproducibility combined with a good validity was found for the novel method using the WBB for measuring isometric lower limb strength in older adults. Further research using the WBB for assessing lower limb strength should be conducted in different study-populations.

## Background

The succesful completion of daily activities such as standing up from a chair or getting out of bed requires an individual to possess sufficient muscle strength[1]. Reduced muscle strength at midlife increases the risk of disability by two to three fold 25 years later[2]. In addition, lower limb weakness has been recognised as a major risk factor for fall accidents in older adults[3]. More specifically the best predicter of being a faller has been found to be reduced isometric whole-lower limb strength[4]. Decreased muscle strength in the lower limb is also seen in patients with stroke[5], knee alloplastic surgery[6], and arthrosis[7] and has been linked to all-cause mortality[8]. Thus the assessment of muscle strength is important both in clinical settings and research as it allows patients, therapists, and researchers to track progress in rehabilitation and adjust exercise interventions accordingly.

Muscle strength or performance is measured in one of four ways: (a) as the maximum force which can be produced during an isometric contraction, (b) as the maximal load, which can be performed once during weight-lifting (eg. squat), (c) as the peak torque during a concentric or eccentric isokinetic contraction, or (d) as a function test (eg. chair-stand test). Isokinetic and isometric dynamometry are considered the gold standard methods for strength assessment[9], [10]. However, these types of dynamometers are often expensive, stationary, and cumbersome to operate and setup, thereby preventing widespread use in standard clinical settings. A common way of assessing muscle strength in clinical settings is manual muscle testing (MMT) using a subjective scale from 0 to 5. However, despite the widespread use of MMT, limitations such as “ceiling effects” and low responsivness to change limit their usefulness[11–13]. In 1965 Beasley showed that an improvement of 50% in the knee extensors monitored by quantitative methods could not be detected by the MMT[11]. Another limitation of lower extremity MMT is poor inter-tester reproducibility with intra-class correlations coeffients (ICC) as low as 0.63[14]. Another relativly simple, valid and low-cost method for measuring muscle strength is hand-held dynamometry (HHD). HHD has been tested for intra- and inter-tester/day reproducibility in different study populations and muscle groups with varying results depending on the muscle groups tested. Reported ICCs have varied between 0.49 and 0.99, coefficient of variation (CV)/standard error of measurement (SEM) have varied between 8–15%, and minimum dectecable change (MDC)/Limit of agreement (LOA) have varied from 19% to 57%[15–18]. In general, imprecision with HHD is greater in stronger patients- and muscle groups as it can be difficult to stabilize the patient sufficinetly[9], although the introduction of belt-fixation has improved the reproducibility of the method[16], [19]. Finally, with HDD it is hard to assess the whole-lower limb strength, which may be a key outcome in terms of discriminating older fallers from nonfallers[20].

Therefore, a need exists to find alternative, reliable, easy to use and accessible methods for measuring muscle strength, which can be applied across different settings. This alternative could potentially be the Nintendo Wii Balance Board (WBB) as it is portable, inexpensive (approximately 80 $), durable[21] and widely available worldwide with more than 43 million boards already sold. Presently only the accuracy of the WBB to measure static forces has been established in a laboratory setting[21]. Therefore, we developed a utility (software and hardware), which allows the WBB to record isometric whole-lower limb strength. To our knowledge, this has not been reported previously. The aim of this study was to explore (1) reproducibility and (2) validity of this novel approach.

## Methods

### Design

The reproducibility of the WBB was tested with an intra-rater inter-day design with seven days between the two test sessions. Concurrent validity was also explored by comparing the WBB against a stationary isometric dynamometer ((SID)—Leg Force, Newtest, Finland). The study followed guidelines for reporting reliability and agreement studies (GRRAS)[22].

### Study-population

Participants were identified and recruited via member lists from senior citizen clubs and organizations in the Aalborg area. Thirty older adults were screened for eligibility and enrolled into the study by using telephone interview. Participants were included in the study if they were 65 years or more, willing and capable of coming to the hospital by themselves twice within a 7 day span, and could pass a small custom cognitive impairment screening (answering the current year, month and prime minister of Denmark). In addition, participants were excluded if they had acute illness within the previous 3 weeks, neurological disease (such as severe dementia, Parkinson’s disease), or had orthopedic surgery within the previous 6 months on either upper or lower extremities. The study was approved by the Danish North Jutland regional ethics committee and all participants gave written informed consent.

### Calibration of WBB and SID

**Fig 1:**
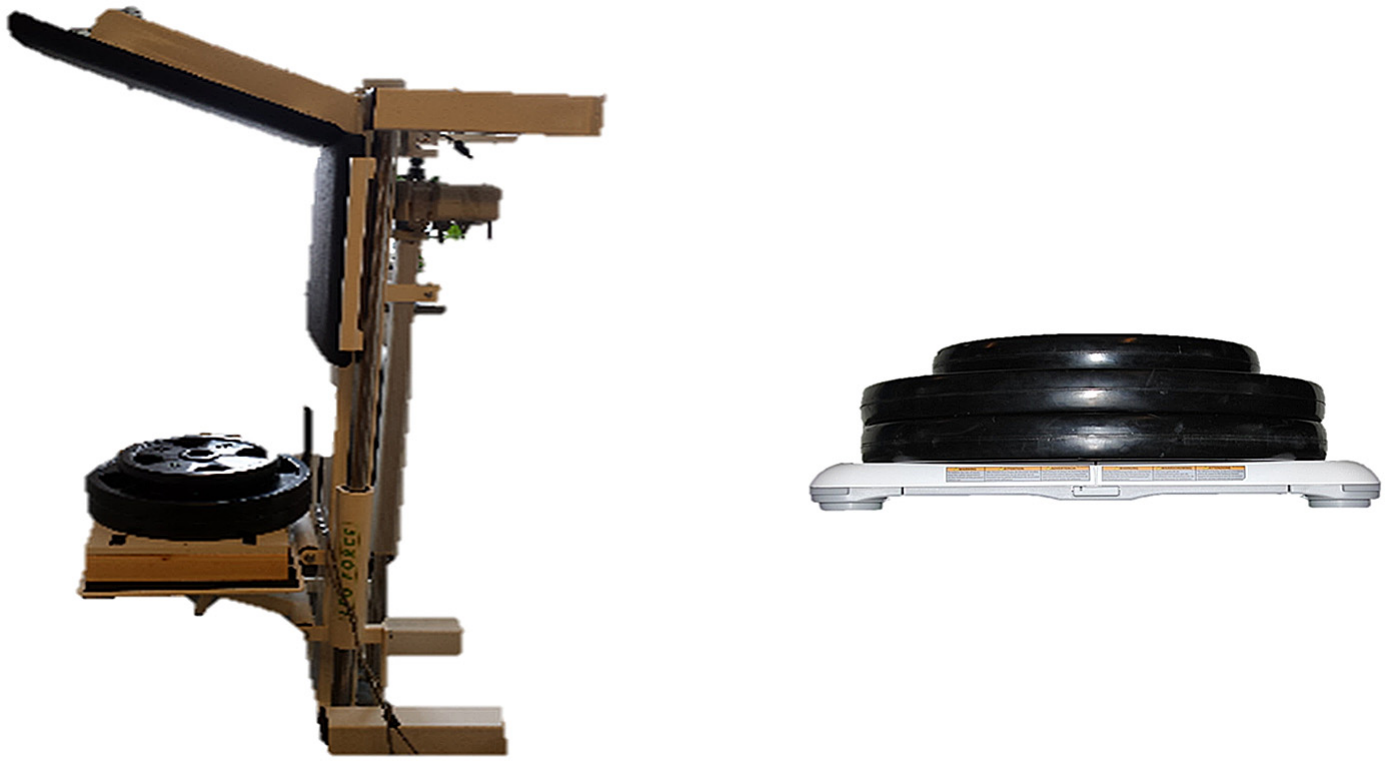
Still pictures of the calibration setup for the SID (left) and the WBB (right).

We loaded both the WBB and the SID with 12 known weights ranging from 25 to 300 kg (adding 25 kg each time) in order to calibrate the measured force values in both evaluation methods. Each mass was centered on both the WBB and the SID “Fig 1”, and the output from the software was recorded. The SID and WBB deviated from the actual weight with 0.9% and 1.4% respectively across the entire tested span. These measured deviations were subsequently accounted for in the respectively software written for both the WBB and the SID.

### Overall experimental set-up and procedures

All participants preformed a standardized warm-up for 5 minutes of sub-maximal cycling with a cadence of 60 rmp with a one kg load. After the initial warm-up participants were randomly (coin toss) assigned to begin on either the WBB or the SID. In the assigned apparatus, participants then performed 3–5 submaximal presses, which served as both warm-up and habituation to the actual strength tests. All tests were performed in a clinical examination room at the university hospital during the same time-of-day and without shoes. Prior to actual testing participants received standardized instructions, which were to “press the feet on the WBB or SID as hard and as fast as possible until being told to stop” in order to ensure that maximal force was achieved. Both the WBB and SID test was initiated verbally with a countdown “3,2,1 Push, push, push”. During both tests (WBB and SID) participants were allowed visual feedback on the force produced as this has been shown to effect the outcome[23]. Between individual recordings, participants were allowed a rest-period of 30 seconds to avoid fatigue. In total three trials were recorded from the WBB and SID in each session and used for further analysis. In this analysis the first recording, mean of the two first recordings, mean of all three recordings and the maximum value of the three recordings were used. Data was collected in the period between mid-November 2014 through mid-December 2014.

### WBB

**Fig 2:**
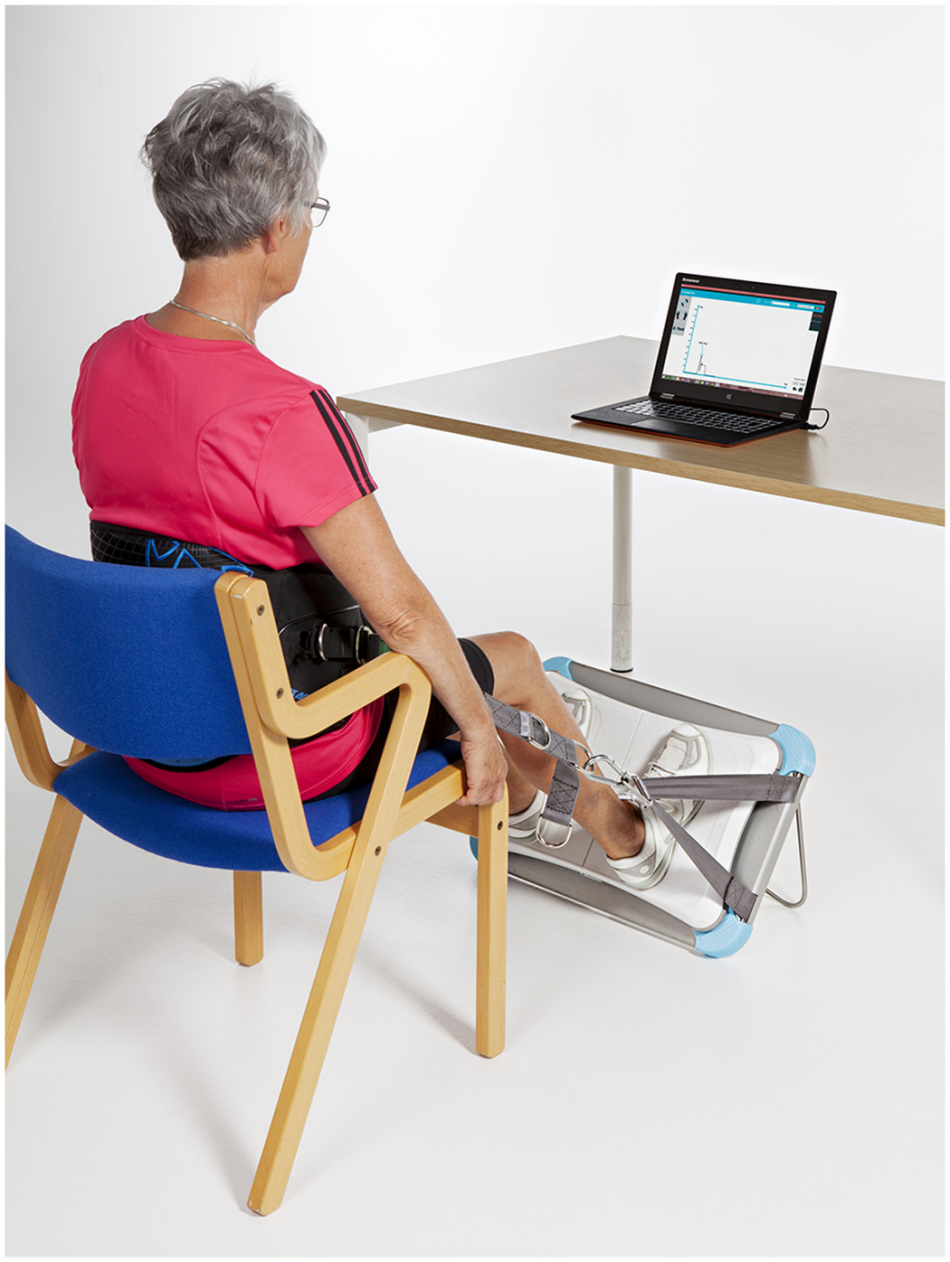
Experimental set-up for the WBB test.

The WBB consists of a rigid platform with four uni-axial vertical stain gauge transducers located in the corners of the board. In order to collect data from the WBB data was streamed to a laptop computer (Lenovo Yoga Pro, Windows 8) using Bluetooth HID wireless protocol and custom programs written in C#. Sensor values were reported as four channels of 16-bit digital data sample at approximately 100 Hz. The signal was subsequent digitally filtered using a 4^th^order Butterworth filter (cut-off frequency 20 Hz). The custom software recorded the isometric force-time curve from the lower extremities during a 20-second period and kilogram was the unit of measurement. Participants were fitted with a standard kite harness (mystic star, size: XL) around their hips, seated in a standard chair (seat height 45 cm), and connected to the WBB via custom seatbelt straps. The seatbelt straps were adjusted in length corresponding to a knee angle of 120 degree. The WBB itself was held at a 57-degree angle from the ground in a custom designed Aluminum-mount. This particular angle was chosen as pilot studies indicated that the 57-degree angle was the angle where most individuals could apply the highest pressure. Finally, participants were allowed to grab on to the side of the chair in order to stabilize themselves “Fig 2”.

### SID

**Fig 3:**
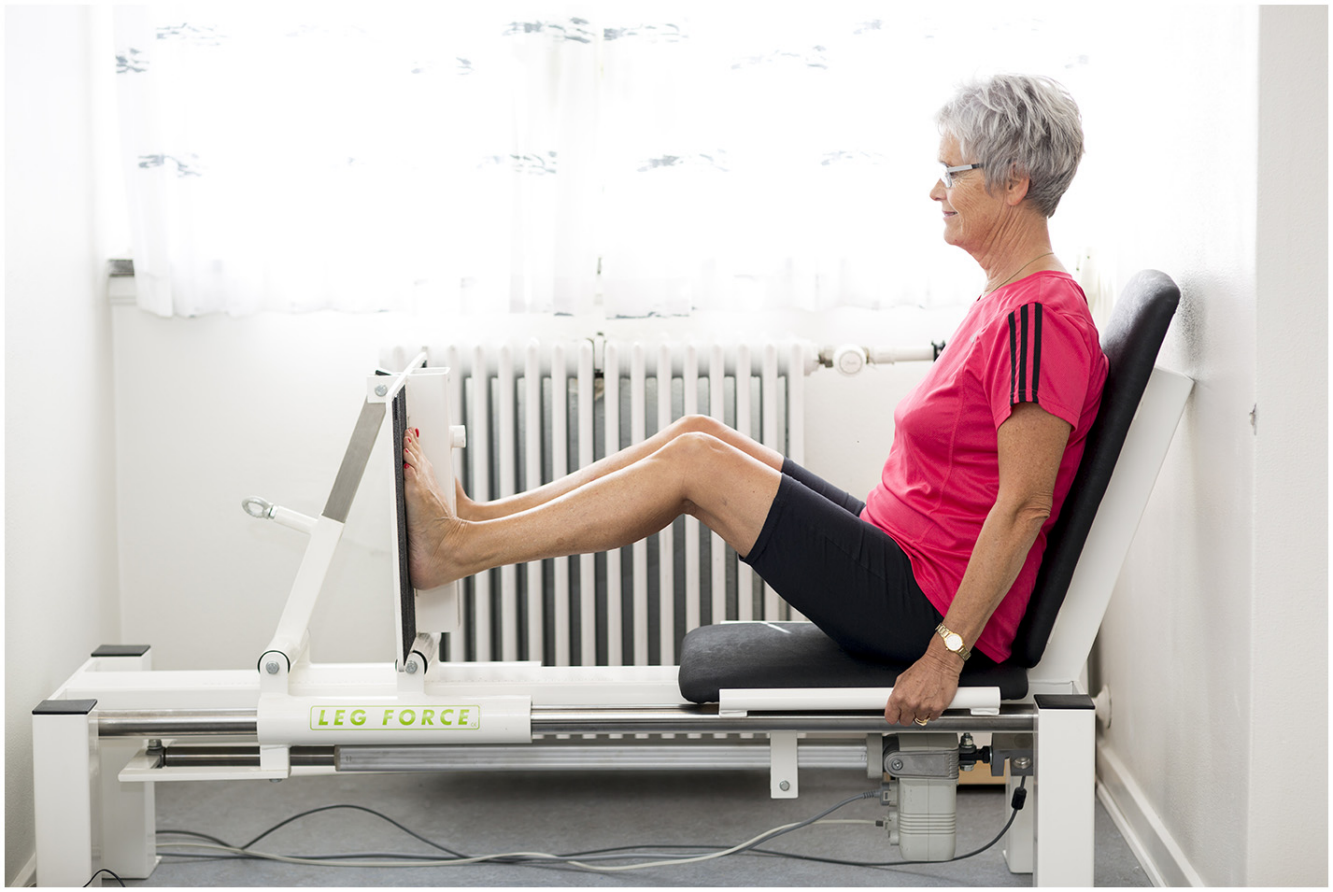
Experimental set-up of the SID test.

Participants were seated in a laboratory-grade stationary isometric SID (Leg Force, Newtest, Finland) with a knee angle of 120 degree and with their feet against a fixed strain-gauge instrumented footplate, while holding on to handlebars (with their hand) for stabilization “Fig 3”. Data from the SID was recorded at 1000 Hz for the duration of the test. The analogue strain-gauge signal was sent through an amplifier and subsequently digitally converted at 1 KHz using a 14-bit, 8-channel A/D converter before being transferred to a Personal Computer. During subsequent off-line analysis, the signal was digitally filtered using a 4^th^ order Butterworth filter (cut-off frequency 20 Hz) (Matlab 7.13, Mathworks, USA).

### Statistics

Data is presented as mean ± standard deviation. The differences between session one and two demonstrated a normal distribution both visually (histogram) and statistically (Shapiro-Wilk), and parametric statistics were applied. Further, no sign of heteroscedasticity was found after plotting each participant’s difference score of the two sessions against the mean of the two sessions[24]. Paired *t* test was used to explore systematic bias between sessions. Relative reproducibility was assessed through an ICC 3.1 two-way mixed model using absolute agreement and the results of a single measurement. The ICC values were reported with a corresponding 95% confidence interval (95% CI)[25]. The correlation coefficients results were interpreted based on ranges of poor (<0.69), fair (0.70–0.79), good (0.80–0.89), and high (0.90–1.00)[26]. Absolute reproducibility was expressed as the standard error of measurement (SEM), and was calculated from the SD of the individual differences between sessions multiplied by $\sqrt{1-ICC}\;$[27]. Limits of Agreement (LOA) were calculated as the SD of the individual differences between sessions multiplied by 1.96. Both the SEM and LOA were also presented as a percentage by dividing SEM and LOA by the average score of session one and two. Validity between the WBB and SID was examined by a Bland-Altman plot (BAP) and ICC. All statistical analyses were performed using SPSS (version 21, IBM, New York, USA).

## Results

Thirty older men (40%) and women (60%) (Mean age 69.0 (4.2)) participated in the study. The characteristics of the participants were height in centimeters 168.5 (6.9), weight in kilograms 72.5 (13.7), BMI 25.5 (4.2), medical preparations 1.5 (1.7), and physical activity in hours per week 8.1 (3.5).

**Fig 4:**
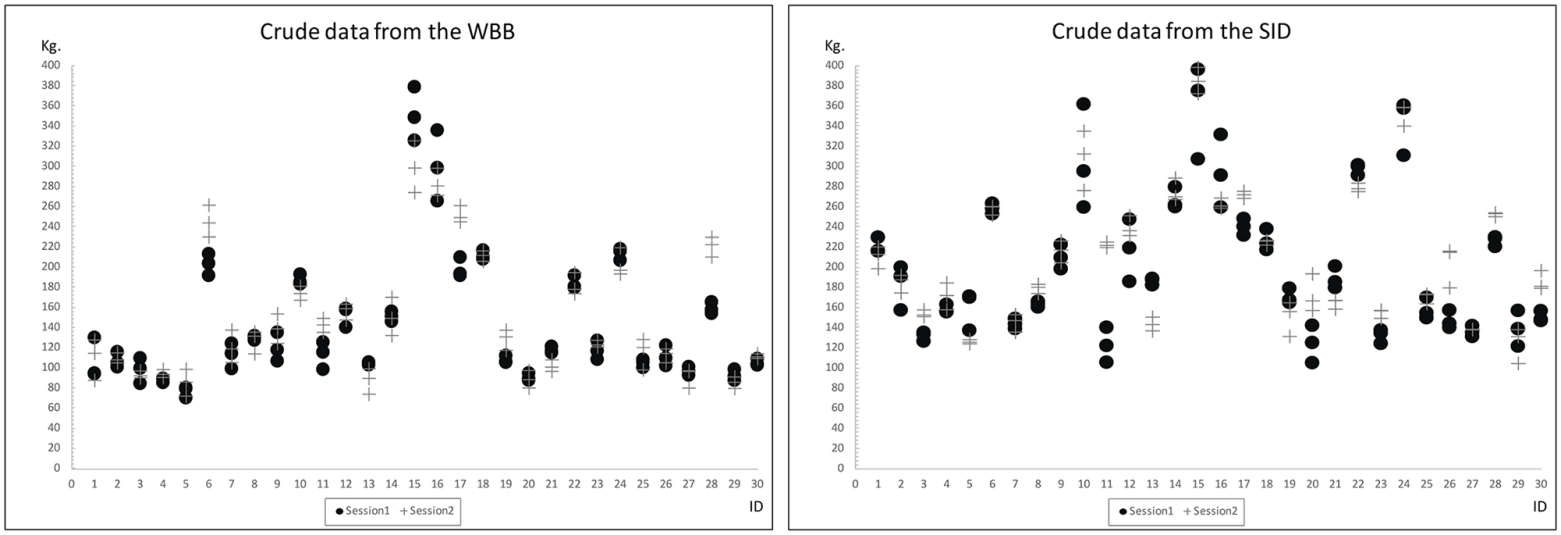
Crude data from the WBB (left) and the SID (right). The horizontal axis shows each individual and the vertical axis show the recorded strength in kilograms for each session.

The crude data from the WBB and the SID are shown in Fig 4. Each mark represents one measurement with “dots” being session one and “+” being session two (subsequent week). The difference between individuals is larger than the difference between measurements within individuals. In addition, the difference between weeks is smaller than the difference between individuals. The results of the WBB and the SID show similarities “Fig 4”.

Systematic bias, relative and absolute reproducibility for the WBB and SID are shown in Tables 1 and 2, respectively. The ICC values tend to be higher and SEM and LOA lower for the WBB compared to the SID. For the WBB the ICC values ranged between .904 and .967, SEM % between 9.7 and 13.9, and LOA % between 20.3 and 28.7. For the SID the ICC values ranged between .904 and .923, SEM % between 11.9 and 13.1, and LOA % between 24.2 and 26.6.

**Table 1:** Results for the Wii Balance Board (WBB) presented as mean, standard deviation (SD), difference between means (M-DIFF), p-values, Interclass Correlation Coefficient (ICC) values with corresponding 95% confidence intervals (95% CI), and Standard Error of Measurement (SEM) and Limits of Agreement (LOA, both in absolute numbers and percentage).

Wii	Session 1		Session 2								
RECORDINGS	MEAN	SD	MEAN	SD	M-DIFF	P	ICC (95% CI)	SEM	SEM%	LOA	LOA%
1	125.9	48.0	132.3	44.3	-6.4	0.078	0.911 [.818-.957]	18.0	13.9	37.0	28.7
Mean of 2 recordings	133.5	47.1	138.0	46.6	-4.5	0.091	0.952 [.900-.977]	13.1	9.7	27.6	20.3
Mean of 3 recordings	138.1	50.8	141.2	49.2	-3.1	0.254	0.957 [.913-.979]	13.5	9.7	28.4	20.4
Max of 3	155.8	66.1	159.3	62.1	-3.5	0.264	0.967 [.931-.984]	15.6	9.9	32.4	20.6

**Table 2:** Results for the SID presented as mean, standard deviation (SD), difference between means (M-DIFF), p-values, Interclass Correlation Coefficient (ICC) values with corresponding 95% confidence intervals (95% CI), and Standard Error of Measurement (SEM) and Limits of Agreement (LOA, both in absolute numbers and percentage).

SID	Session 1		Session 2								
RECORDINGS	MEAN	SD	MEAN	SD	M-DIFF	P	ICC (95% CI)	SEM	SEM%	LOA	LOA%
1	197.4	63.1	210.2	72.7	-12.8	0.017	0.904 [.784-.956]	26.8	13.1	54.3	26.6
Mean of 2 recordings	202.0	64.5	212.0	67.3	-10.1	0.056	0.904 [.801-.954]	26.7	13.0	54.4	26.3
Mean of 3 recordings	204.7	65.9	212.5	67.4	-7.9	0.106	0.921 [.840-.962]	24.8	11.9	50.5	24.2
Max of 3	221.2	74.9	224.1	73.5	-2.8	0.600	0.923 [.846-.963]	28.5	12.8	57.6	25.9

**Fig 5:**
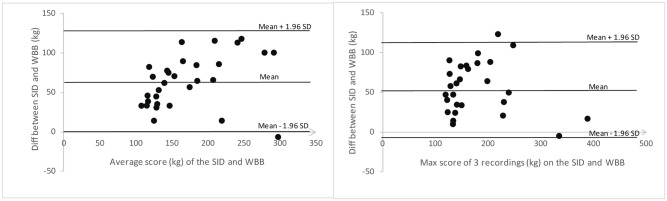
Bland-Altman plots representing comparisons between the SID and the WBB on the first occasion using (A) a mean of three recordings and (B) the maximum of three recordings. The mean line represents the mean difference between the devices, with the upper and lower lines representing the limits of agreement (1.96 SD).

The between-device ICC values were .809 (95%CI .637-.904) and .840 (95% CI .690-.920) using the mean and maximum value of three recordings on the first session, respectively. The BAP did not show a relationship between the differences and the mean values “Fig 5”. However, it did show that the WBB differs on average approximately by 60 kilograms from the SID.

## Discussion

The ability to objectively assess whole-lower limb strength using a portable, affordable, quick, reproducible and valid system could provide numerous benefits in a number of populations. The present study showed that measurements with the WBB could be performed without systematic bias, a high relative reproducibility, and an acceptable absolute reproducibility across different days. In addition, we found that WBB had a good concurrent validity with the SID. These results encourage and facilitate further research into clinical applications in different study-populations using the WBB for measuring lower limb muscle strength.

To our knowledge, this is the first reported study to explore the use of a WBB to assess whole-lower limb strength. HHD and SID are commonly used methods and are quite similar to our method. These methods were an obvious starting point for evaluating the external validity of our novel method. The present method showed similar or better relative and absolute reproducibility compared to previous reports for isometric strength in the knee extensors using either SID or HHD. Previous studies showed ICC’s between .49-.99, CV/SEM between 8–15%, and MDC/LOA between 19 and 57%[15–18]. The reported ICC’s in the present study for the WBB were high (>.911) and clinically acceptable for both SEM (9.7%) and LOA (20.3%) when averaging two recordings. In addition, the WBB outperformed the SID with a slightly better relative (ICC 952 vs. .904) and absolute (SEM 9.7% vs. 13%; LOA 20.3% vs. 26.3%) reproducibility using the average of two recordings. This supports a potential use of the WBB in both research and clinical settings.

Despite fairly high and acceptable levels of reproducibility, random variation still persists the cause of which is unclear. Potential reasons include biological (both within the participant and between participants), instrumental, and/or experimental protocol variations? In the present study, we believe the main contributors to variation were biological and caused by the experimental protocol. This notion is supported by an unpublished laboratory sub-analysis that we have previously performed, which showed that instrument variation was less than 0.1 kilogram when we did repeated tests (10 trials) by placing known weights (15, 50, and 100 kg) on the WBB. This supports the idea that variation is caused by biological and/or experiment protocol reasons, and emphasizes that future studies and/or clinical use should focus on minimizing these types of varations.

Within the community of researchers and clinicians a general agreement seems to exist that reporting several statistics of reproducibility (both relative and absolute) is appropriate [22], [25], [27]. The current study followed this guideline in an attempt to report the method in a transparent way to the reader. However, this transparency has not been the common standard within reproducibility studies in the last 15 years as several methods have been deemed reproducible, while only reporting relative reliability such as ICC[28–30]. The ICC analysis has certain limitations such as being very sensitive to variation between individuals. As an example this means that the chance of obtaining a high ICC increases when individuals differ from each other (heterogeneity). Therefore using ICC (relative reproducibility) as a sole measure to conclude a method is reproducible is not appropriate. Of absolute outcomes, we chose to calculate SEM and LOA, and not CV as we found data to be homoscedastic. Our choice of reporting both SEM and LOA stems from an ongoing discussion within the literature of reproducibility, concerning what confidence level absolute measures should be reported at (68% or 95%?). SEM, CV, and Typical Error all report limits of error with a 68% probability. LOA, smallest real difference (SRD) and minimum detectable change (MDC) provide confidence of error at a 95% probability level. This means that the measurement error between test-retest is predicted to lie within the limits of agreement with a 95% probability when testing a new individual from the study-population. In practical terms, this means that one can be 95% certain that a true change has occurred when the effect of rehabilitation exceeds the LOA mark. With the above in mind, experts have suggested that the 95% probability is a very conservative level, and that an appropriate probability level should be half the limit of agreement [25]. This is further supported by the notion that most observations will lie within one standard deviation from the mean difference, given that the standard deviation of differences between tests follows a normal distribution. In the current study, we found the SEM of the WBB to be 9.7% and the LOA to be 20.3%. If we accept, a probability level halfway between the 68% and 95% level, then the present method would have a measurement error of approximately 15%. A difference of 15% or more has been reported in studies of lower limb extensor strength in a number of populations [5], [6], [31–33]. This indicates that the current method with a SEM of around 10% and LOA of around 20% has the capability to detect “true changes” following rehabilitation.

The present protocol for measuring lower limb muscle strength had limitations. Firstly, the seamless adjustment of the belt between the harness and the aluminum-board meant that the tester had to measure the angle of the knee (120 degrees) each time and adjust the belt accordingly. This factor (experimental protocol) may be one of the largest modifiable contributors to within-subject variation, as the tester needed to be cautious on the exact length of the belt between test and retest. It may be more appropriate to work with three given lengths of the belt, i.e. long, middle and short, adjusted based on the subject’s leg length (trochanter major to the floor). This modified assessment protocol would limit the influence of belt length as a source of within- subject variation. Secondly, the study lacked between-tester data on the method. These data could be relevant for further validation. On the other hand, we would not expect any significant difference between testers as the present method is objective, straightforward and well described. Finally, the present method did not dictate another recording if the last obtained strength value was the highest in the present session. Continuing to record additional values until a “ceiling” had been reached could have decreased the within-subject variation. A strength of the present study was the use of both relative and several absolute statistics. This transparency in reporting should help the reader to better judge the reproducibility and provide basis for comparisons with other studies.

In summary, the novel approach using the WBB for measuring isometric lower limb strength in older adults showed a high relative reproducibility, an acceptable absolute reproducibility and a good concurrent validity. The results encourage further research using the WBB for muscle strength testing in different study-populations.

## Supporting Information

S1 Dataset (XLSX)
